# Primers for complete chloroplast genome sequencing in *Magnolia*

**DOI:** 10.1002/aps3.11286

**Published:** 2019-09-19

**Authors:** Eunji Song, Suhyeon Park, Sangtae Kim

**Affiliations:** ^1^ Department of Biology Sungshin University Seoul 01133 Korea

**Keywords:** chloroplast genome, Magnolia, Magnoliaceae, Sanger sequencing

## Abstract

**Premise:**

A new set of primers was developed for sequencing of whole chloroplast genomes of *Magnolia* species and gap‐filling of unfinished genomes.

**Methods and Results:**

Two hundred and fifty primers were newly designed based on two previously reported chloroplast genomes from two different genera in Magnoliaceae. A total of 134 primer pairs, including the ones developed in this study and 18 previously reported ones, were enough to cover the entire chloroplast genome sequences in Magnoliaceae. Four species from different sections of *Magnolia* (*M. dealbata*,* M. fraseri* var*. pyramidata*,* M. liliiflora*, and *M. odora*) were used to show the general application of these primers to chloroplast genome sequencing in *Magnolia*.

**Conclusions:**

Using the developed primers, four *Magnolia* chloroplast genomes were successfully assembled. These results show the utility of these primers across *Magnolia* and their potential use for phylogenetic studies, DNA barcoding, and population genetics in this group.

The family Magnoliaceae is characterized by the presence of (1) numerous stamens and carpels that are spirally arranged on an elongated floral axis, and (2) an undifferentiated perianth (except for some species in *Magnolia* L. section *Yulania* (Spach) Dandy) (Figlar and Nooteboom, 2004). In this family, 298 species are distributed mainly in Southeast Asia (ranging from India to the Kuril Islands including New Guinea) and the Americas (ranging from eastern Canada to Brazil including the Caribbean) (Govaerts et al., 2017). The current classification system of Magnoliaceae includes only two genera, *Liriodendron* L. with only two species and *Magnolia* comprising 296 species divided into three subgenera and 12 sections (Figlar and Nooteboom, 2004). A comprehensive phylogenetic study using 10 chloroplast regions (both genes and intron/intergenic spacers) suggests 12 major clades in Magnoliaceae with a basal polytomy in *Magnolia* (Kim and Suh, 2013).

The reliability of phylogenetic inferences is heavily dependent upon the number of phylogenetically informative characters (Dong et al., 2013). To elucidate the relationships among major clades in *Magnolia*, a comparative genome analysis that provides more phylogenetically informative characters is needed. The chloroplast genome sequence is an essential resource in the study of plant phylogeny, and several approaches have been suggested for the completion of chloroplast genome sequences. Currently, next‐generation sequencing–based genome skimming is commonly used for the de novo assembly of chloroplast genomes. Although techniques such as organelle isolation, hybrid capture, and methylation enrichment have been developed to improve the efficiency of this work, there are still challenges in the completion of chloroplast genome sequences, particularly for genomes assembled from herbarium material or for structurally divergent genomes (Twyford and Ness, 2017). In some cases, assembly using next‐generation sequencing data generates incomplete genomes and critical parts of the assembly need to be resequenced. Therefore, short‐range PCR in combination with traditional Sanger sequencing is still used as an alternative, complementary method to assemble complete chloroplast genomes (Dong et al., 2013). For example, a set of universal primers designed in Saxifragales was successfully applied in the phylogenetic study of that family (Dong et al., 2013).

In this study, we report and test 134 sequencing primer pairs to cover entire chloroplast genomes in *Magnolia*. These primers can be used for de novo sequencing or finishing incomplete chloroplast genomes, as well as for phylogenetic, DNA barcoding, and population genetic studies in Magnoliaceae. Additionally, these primers will be a useful resource for chloroplast microsatellite development. The utility of chloroplast microsatellites in Magnoliaceae has been well demonstrated by Kuang et al. (2011).

## METHODS AND RESULTS

**Figure 1:**
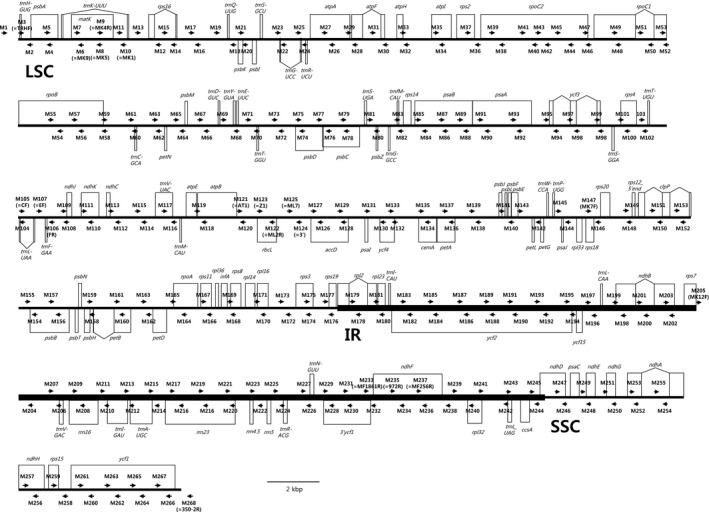
Sequencing primer positions (arrows) along the linearized chloroplast genome map of *Magnolia kobus*. One inverted repeat region is not shown. The genes above the line are transcribed in the reverse direction, whereas the genes below the line are transcribed in the forward direction. IR = inverted repeat; LSC = large single‐copy region; SSC = small single‐copy region.

We designed 116 pairs of csly reported chloroplast genomes in Magnoliaceae: *M. kobus* DC. (Song et al., 2018; NC_023237) and *L. tulipifera* L. (Cai et al., 2006; NC_008326). These sequences were aligned using CLUSTALW (Higgins et al., 1994), and primers were designed in the shared sequence regions of two chloroplast genomes using Primer3 (with default settings; Untergasser et al., 2012) or OLIGO (version 5.0; National Biosciences Inc., Plymouth, Minnesota, USA) (Table 1). PCR products generated from these primers along with the previously reported 18 primers (Kim and Suh, 2013 and references therein) covered the entire chloroplast genome in Magnoliaceae (Fig. 1). Four species from different subgenera and sections of *Magnolia* (*M. dealbata* Zucc., *M. fraseri* Walter var*. pyramidata* (W. Bartram) Torr. & A. Gray, *M. liliiflora* Desr., and *M. odora* (Chun) Figlar & Noot.) were used to determine the broad applicability of these primers to chloroplast genome sequencing in *Magnolia* (Appendix 1).

**Table 1:** Primer pairs used for chloroplast genome sequencing in *Magnolia*

Primer pair	Forward primer^a^		Reverse primer^b^		Size in *M. kobus* (bp)	*T*_a_ (°C)	PCR success^c^			
							Mde	Mfr	Mli	Mod
1	M1	ATAAGCCAGATGACGGAACG	M2	CATTTCTTCCTAGCCGCTTG	1417	55	+	+	+	+
2	M3 (=TRHF^d^)	CGCATGGTGGATTCACAATC	M4	GCCCTTGGATTGCTGTTG	1077	55	+	+	+	+
3	M5	AGGCATACCATCAGAGAAGC	M6 (=MK9^d^)	CTTCGACTTTCGTGTGCTAG	1314	55	+	+	+	+
4	M7	ATCCAAATACCAAATCCGTT	M8 (=MK5^d^)	CACTGCTGGATACAAGATGC	990	52	+	+	+	+
5	M9 (=MK4R^d^)	TTTACGGAGAAACACTAATACG	M10 (=MK1^d^)	ACGAATGTGTAGAAGAAACGG	960	55	+	+	+	+
6	M11	CCTCTCTCTTTCCATCCAAT	M12	GGGGGCATTGTTCATCTA	1503	55	+	+	+	+
7	M13	AAGAGATTGGATTGCCCTAC	M14	AGGGTTAGTGCCAGTCAATA	1450	55	+	+	+	+
8	M15	GCCGTCTCTAACCTCTTTTG	M16	CGACTTGTTGATTTGATTGATT	1211	55	+	+	+	+
9	M17	CGGAAAAGTCGCAAGTGA	M18	GGTTTTGGTCCCGTTACT	1570	52	+	+	+	+
10	M19	CACCCCAGTCTTAGGAGC	M20	CAAACAAGGGCTAAGAGAAA	1083	55	+	+	+	+
11	M21	TTACCCGAGGCTTATGCT	M22	CGAAAGACCCCCTAACTATT	1590	52	+	+	+	+
12	M23	TATGTTCCGACTTCAATGGC	M24	GTTTCATTCGGCTCCTTTAT	1056	55	+	+	—	+
13	M25	CTCCCTTTTTCCATACATCG	M26	GCTTATCGCCAAATGTCTCT	1262	55	+	+	+	+
14	M27	GGAGACGGAAATACCCACAT	M28	CGAGTTACATTTACGCACCA	1235	55	+	+	+	+
15	M29	TTCCCCTGCCATTACTTC	M30	GAGTGTGTGCGAGTTGTGTATT	1350	52	+	+	+	+
16	M31	GCGAGACACCCATTTTTC	M32	GCTTGCTTCTATTGGACCTG	1184	55	+	+	—	+
17	M33	CCATAAAAGCCAGACTAAGC	M34	CAACCAACCCCAATACTTTTAC	1514	55	+	+	+	+
18	M35	AATCCCGCTTGTGAATAATC	M36	GCAGGAGTTCATTTTGGTCA	1554	52	+	+	+	+
19	M37	TTTCCCCGTCTTTTGTTC	M38	GAAAAGAGGATTGAAGGTTG	1276	52	+	+	+	—
20	M39	GATGCCCTCGTTATTCCC	M40	GGAATCAAAAAAATGGAAAAAT	1557	51	+	+	+	+
21	M41	GGCATTCCTTATTTCTATTCAG	M42	GAAAGAACTAATGCCCCG	1034	52	+	+	+	+
22	M43	CGGGAATGAAAAAAAATCG	M44	CTGTAGATTATGTTATGGTCGG	1112	51	+	+	+	+
23	M45	GCGAATCTCAGCAATCACTT	M46	GCCACTGCTACATCCATTTC	1122	55	+	+	+	+
24	M47	TGTTGTTCAGCATCTTGGAC	M48	CATTTGTCATTCGTGGTCTA	1215	52	+	+	+	+
25	M49	GGTGGGTGCTCTATTCAG	M50	ATTAGCCATTCCATTTCTTTTA	1440	52	+	+	+	+
26	M51	ACACCAAATAAAGAAAGGGG	M52	GGAGAAGTGACAAAACCCTA	988	52	+	+	+	+
27	M53	CGACCCCGCATTGTTCAC	M54	CGAACACGAGGGAAAGAT	1774	52	+	+	—	+
28	M55	ATGCGGTATTTCGTTAGTGA	M56	ATTGGCTCTGGTTCGTTTAG	1156	52	+	+	—	+
29	M57	TTGAGATAAAGGGTGTAGGC	M58	GATGGAAATGAGGGAATGTCTA	1119	55	+	+	+	+
30	M59	CAATGAACCTACAAAATCCCTC	M60	CCAAAACAAAAAGAAATCCC	1193	52	+	+	+	+
31	M61	TTTTGGATTCTGTAACTGGA	M62	CATTCTTGGCGGGGTTAC	1015	52	+	+	+	+
32	M63	ATTGGATGGGTGATTGGC	M64	TCCATTTGTATTGATTCCGA	1048	52	+	+	+	+
33	M65	TACAATGAGGAGCAACCAAC	M66	TTTCTTCCTATTTTACCCCATC	1113	55	+	+	+	+
34	M67	CTCATTTCCACTCTTTCTTTTC	M68	GTCTACGCTGGTTCAAATCC	1399	55	+	+	+	—
35	M69	GTGCTCTGACCGATTGAACT	M70	TAGGGGGCTCATTCAAGA	1274	52	+	+	+	+
36	M71	AACTCGTAAATCTGGGAAGG	M72	CTTTCTCGCATTCGCTCT	1244	52	+	+	+	+
37	M73	TTTATTCCGAGTCACAAGAGC	M74	GCGAAATAAGCACAAGGAAA	1022	52	+	+	+	—
38	M75	TTCGGAAATGGTTGAAGTAG	M76	TGATAAGTCGGGCATTCC	1177	52	+	+	+	+
39	M77	CGGTTTATGGATGAGTGCTA	M78	GCGATGAAACCAAAGACAGA	1033	55	+	+	+	+
40	M79	GGGGAGAAGGATGGATTG	M80	ATTCCCACTTTATTTTTATTCG	1303	52	+	+	+	+
41	M81	ATCTCTATTTTATTCCCCCG	M82	TTCGTCCATTAGTTCTCAGTTC	1156	52	+	+	+	+
42	M83	CCTCCTCTTTTCCTCCCA	M84	CTTGTTTGGGCTACTGGATT	983	55	+	+	+	+
43	M85	GTAGAGGCAATCAAGAAAGC	M86	ATCACCAATACATCGCAGGA	1066	55	+	+	+	+
44	M87	GAACCCCAGAAACAGGCT	M88	CAATCGGCTTACGCACTA	917	52	+	+	+	+
45	M89	TCGGCATTTTTGAACCAC	M90	GCAGTCAGATGTTTGGGG	958	51	+	+	+	+
46	M91	CACCCAGGAAAAAAAGGC	M92	GCTTTTTGCTGGTTGGTT	1511	51	+	+	+	+
47	M93	CTCGGCAAAACTGGGATA	M94	ATTGACCCACCTATTCCG	1622	52	+	+	+	+
48	M95	TACCAGATGAGATAGAACGATG	M96	CAACGGAGAACATACGAAGG	1137	55	+	+	+	+
49	M97	TCGGCTCGTATGAAGTCTCT	M98	GAGATGGTGCGATTTGATTC	1125	55	+	+	‐	+
50	M99	GGGATACACGACAGAAGGAA	M100	GACTTTTCACTCATCCCAAT	1195	52	+	+	+	+
51	M101	CGGAAAGAGTGGAAAAGAAT	M102	ACAGAACAAATCAAGAAAAGGA	955	52	+	+	+	+
52	M103	CTGAACTAAACGATAAACGAAG	M104	CAATCCAATCAAGTCCGTAG	1190	55	+	+	+	+
53	M105 (=CF^d^)	CGAAATCGGTAGACGCTACG	M106 (=FR^d^)	ATTGAACTGGTGACACGAG	987	55	+	+	+	+
54	M107 (=EF*)	GGTTCAAGTCCCTCTATCCC	M108	GGGCTAATAAAAGAAAGGGG	1075	55	+	+	+	+
55	M109	TTTCTATTTCTTTACTCCCTCC	M110	TGGGTCTCAACAGGAAAATC	1043	55	+	+	+	+
56	M111	CACAAACACACCCTGCCT	M112	ATGACCCACAGCAAACAAAC	1250	55	+	+	+	+
57	M113	AATGCCAAAATAGGAATAACAC	M114	GAATCCCCCAACTCATCACT	1230	52	+	+	+	+
58	M115	GGTTAGGCTTCGTGACAATA	M116	GTGCCAAATAGAACCCATCA	1371	55	+	+	+	+
59	M117	TTGACAGGAAGATAACGAGATG	M118	GATGGTCTTCCCGAGCAG	1467	55	+	+	+	+
60	M119	TACGGCTGTGGCAATAGG	M120	TACCAACGAAATCAAGCG	1751	51	+	+	+	+
61	M121 (=AT1^d^)	AGAACCAGAAGTAGTAGGAT	M122 (=ML2R^d^)	TTCAATTTATCTCTCTCAACTTGG	1276	52	+	+	+	+
62	M123 (=Z1^d^)	ATGTCACCACAAACAGAAACTAAAGCAAGT	M124 (=3’^d^)	CGGCTCAACCTTTTAGTAAAAGATTGGGCCGAG	1508	55	+	+	+	+
63	M125 (=ML7^d^)	GGAGGAACTTTAGGACACCC	M126	TCCCTGACACCTAAAAAATGAT	1096	55	+	+	+	+
64	M127	CAAATAGGGGGCAGGAAG	M128	GTTGTAGGAGATGTAAGGATTG	1204	55	+	+	+	+
65	M129	GGTGTGTGCTTCTGGAGGAG	M130	CGTTCGGATTGCCAGTTC	1620	55	—	—	+	—
66	M131	TTACCCTCTATTTTTGTGCC	M132	CGAGTCAAGGGAATGGCT	1017	52	+	+	+	+
67	M133	GTGTGTATTTTTCGTTGGGG	M134	TTATCATTTCGTCCAACAGG	1424	52	+	+	+	+
68	M135	GATTCAAAGTGCCAAAAAAG	M136	ACAGTATCAGGAAGCACAGC	1137	52	+	+	+	+
69	M137	TGGGTAAAGGAACAGATGAC	M138	TATTCTCCTCCTACTTATGCCT	1279	55	+	+	+	+
70	M139	TGTTTTGCTTGCTTTGTTTA	M140	ACCCGAACGAACAAAATG	1439	50	—	+	+	+
71	M141	CTATCAGCCAAAGAGGAATC	M142	TGCTCAGACCAATCAATAGA	1299	52	—	+	—	+
72	M143	GTTCTCCCGTGCTTCCAG	M144	AAAGACCCAAACCATAGAGTAG	1784	55	+	+	+	+
73	M145	ATCCCTGTCTTGTTTTCCAC	M146	CGAACAAAACATCAATCAATCT	1586	52	+	+	+	+
74	M147	CTTTTCGTAGGCGTTTGC	M148	AAGAAGCAGAAAGATTATG	1420	52	+	+	+	+
75	M149	CACACTCTTTGGCTCTACCC	M150	CCTTTTTGCTTCCACACC	1331	52	+	+	+	+
76	M151	GACAAATAGAATCCATCAGACC	M152	GTCGTAGCAAAAAGAAGTGG	1149	55	+	+	+	+
77	M153	TTTTGACTTGACTTGCTTCC	M154	ACAGAAAGCAACCGACCG	1165	52	+	+	+	+
78	M155	CTGCTTCTCTTTGTTCCTACGA	M156	AATAATCCCCCTTTCGCC	1148	52	+	+	+	+
79	M157	GCTTTCGTTGTTGCTGGA	M158	ATAGAGCCATTGCGACAC	1516	52	+	+	+	+
80	M159	CGAACTATTACAGGGGATTT	M160	AAAAAGTCATAGCAAAACCG	1250	52	+	+	+	+
81	M161	CGAGATTCAGGCGATTGC	M162	AGCCTCCGTTCTTCCTTA	1159	52	+	+	+	+
82	M163	GAGGATAGGCTGGTTCGC	M164	TGCGGAGGAACAGGACAT	1578	55	—	—	+	—
83	M165	CTAAGGAAGAACGGAGGC	M166	GGACACCATTTGCTGCTC	2345	55	+	+	+	+
84	M167	CGTCTTTTTTTAGGAGGTCT	M168	TTGGAGGAGAAGTTTTGTGT	1141	52	+	+	+	+
85	M169	TTTTGTTCTTTCATTCCAGG	M170	GAAATGGGCGGAGTATCG	1303	52	+	+	+	+
86	M171	AATGGGTCTGAGGTTGAATC	M172	AAAAGGCAGTGTGATAAAGC	1208	52	+	+	+	+
87	M173	TTGGTTCCTGGTTGGTTC	M174	GCAAAACCTTATGGACAACC	1049	52	+	+	+	+
88	M175	CCTTTTGTATCCGCTTGTTC	M176	GGAGAAGGTGGAAGAAGGTC	989	55	+	+	+	+
89	M177	CTCATAGGAACGCCCACG	M178	ATAAGCCAGATGACGGAACG	1195	55	+	+	+	+
90	M179	ATCAATAAAAACCCCTTCCC	M180	ATCATTACGCTTCAACCG	1109	51	+	+	+	+
91	M181	CGACCTTTACCACAATGATG	M182	CCCCAGTTAGATTCAGGC	1269	55	+	+	+	+
92	M183	TTTGATGGGGCTTCTTCC	M184	TGTCAGAGAAAAAAGAACGAAT	1196	52	+	+	+	+
93	M185	CAAACGGAACGAACAGAG	M186	CCCGATACTCACAAAGAAAA	1362	52	+	+	+	+
94	M187	CCGTTTTCAAGTAGTGTTCG	M188	AGCACTATCTCGTTGAAAGG	1165	55	+	+	+	+
95	M189	ACTTATTGTCAGCCTCTTTCAG	M190	TCTCTTTCTTCATCATCAATCG	1115	55	+	+	+	+
96	M191	CATACCAAATCCCATCAATC	M192	GCAACAGCCCTTCCTATC	1329	52	+	+	+	+
97	M193	GGCTTCTTATTCCACAACAA	M194	TCGGATGGAGTATTAGAACG	1324	52	+	+	+	+
98	M195	CCCTTTGTCTCTGTGTTTTC	M196	GTTTTAGGGATTGGCGAC	1048	52	+	+	+	+
99	M197	TGGATTCTCTTTCGGATAGG	M198	CGAAACCAAGAAATAACCCC	1282	55	+	+	+	+
100	M199	CATAACCCCAGCCCATTC	M200	TTTCTGACTTGCTCCTACGG	1129	55	+	+	+	+
101	M201	GACTTTCATCTCGCACGG	M202	CCGATGGAGAGAAGAACCTA	1191	55	+	+	+	+
102	M203	AGGTAGGAGCATAAACTGAAAC	M204	AAAAGGAGGGAAACGGATAC	1530	55	+	+	+	+
103	M205	CACTTATTTTGGCTTTTTGACC	M206	TGGGATAGGGATAGAGGAAGAG	1391	55	+	+	+	+
104	M207	TTACCAAAATGTGCGGAT	M208	GAAGCAGAACCAAGTCAAGA	1262	55	+	+	+	+
105	M209	AGGCAAGAGGATAGCAAGTTAC	M210	GCCGTGTCTCAGTCCCAG	1243	55	+	+	+	+
106	M211	GGACGGGAAGTGGTGTTT	M212	CGGGTTTTTGGAGTTAGC	1177	52	+	+	+	+
107	M213	TCGTGCCGTAAGGTGTTG	M214	CCGTCACCCCAGAATAAAAG	1208	55	+	+	+	+
108	M215	TCAGGAGGATAGATGGGG	M216	CCGCCGACTCCAACTATC	1157	55	+	+	+	+
109	M217	GCGATTACGGGTTGGATG	M218	GGTTGTCTCTTGCCTGCC	1145	55	+	+	+	+
110	M219	CCTTCCATTTAGCAGCAC	M220	GCATTTTTACATCCCACAGC	1253	52	+	+	+	+
111	M221	GAGACGATGGGGGATAAG	M222	CGCCCCATAGAAACTGTC	1306	55	+	+	+	+
112	M223	GTAAGTTCCGACCCGCAC	M224	TAGAGAGGGAGGGCAGAG	1154	55	+	+	+	+
113	M225	GGGATGGAGCGACAGAAG	M226	GAATCACCGTCAATACCTCG	1268	55	+	+	+	+
114	M227	TTTGTGTTTTACTCCCCG	M228	AGAAATGAAACAAAAGATACGG	1148	52	+	+	+	+
115	M229	CGGACTCTATTATGGATTTCTG	M230	CGAAAAGAAGAGTCACAAGAGG	932	55	+	+	+	+
116	M231	TACCGTCGCCTATTGTCAC	M232	GTCCTATTTACTTTGTTTGTTG	1215	52	+	+	+	+
117	M233(=MF1861R^d^)	TGAAAAGATGAATAAACAGACCC	M234 (=MF561^d^)	TGGTTTATTATTAGGAATCTTAGG	1323	55	+	+	+	+
118	M235 (=972R^d^)	CATAATATAACCCAATTGAGAC	M236	ATCGCCGTAATAGTGGAATG	1298	52	+	+	+	+
119	M237 (=MF256R^d^)	TGGGTCGATCAAGTGGCC	M238	TCTACGAATACGCTTTTTTG	1468	52	+	+	+	+
120	M239	GTAGCGGACCTCATAGACATAG	M240	GTGTGAGGATTTACCGAACC	1250	55	+	+	+	+
121	M241	GACTTTGCTTTGTAACTCTCCG	M242	GACTAATGACACGATAACTCCA	1616	55	+	+	+	+
122	M243	GTGCCTGCTCTACAATCC	M244	TTTTCTCCCTGGTTGATG	1479	52	+	+	+	+
123	M245	CCATTGAGTCCCGTATCG	M246	TGCTCCTGCTCCAAGAAC	1241	55	+	+	+	+
124	M247	ACCAAGGAAAATAACTCGTG	M248	GCCGTGTTTTGTTCTGTGTT	1185	52	+	+	+	+
125	M249	CCGATAGAAAATAAATAGGCAC	M250	GGATAACCCCCTTGATTC	1229	52	+	+	+	+
126	M251	ATCCCGCTTTTGTATCCG	M252	CTTTACTTGGGCGGATGG	1072	52	—	—	+	—
127	M253	ATAGGAATGAACAGGAACAAAT	M254	AGTAAACATAAGCAGTGGAAAC	1284	52	+	+	+	+
128	M255	CGTTCCCGATAGTCATTTCT	M256	AATGGCAAAAAGAAGGAGAC	1527	52	+	+	+	+
129	M257	TCCTTTTGGGGCTTCTACTC	M258	TGACTGGCATTATTATTATTCC	1470	52	+	+	+	+
130	M259	CCAAATGTGAAGTAAGTCTCCG	M260	CACGAAACCGACAAAAAG	1346	52	+	+	+	+
131	M261	ATCCATTGTCCATCCCAT	M262	TGATGAAAGAAATAAAGAAGGA	1638	52	—	—	+	+
132	M263	CTCTATTTCGCCATTTTTGC	M264	GAGGATTGGAAGGAGTGG	1351	52	+	+	+	+
133	M265	TTTTTCCTTTCTTTTTCATTCG	M266	TCAGAAAATCAAACGAAATG	1015	52	+	+	+	+
134	M267	ATTCTTCCTCATTTTCTTGCTC	M268 (=350‐2R^d^)	GGAAGAAAAGGAGGATCCGG	1001	55	+	+	+	+

Note

— = unsuccessful amplification; + = successful amplification.

a Primers above the line in Figure 1.

b Primers below the line in Figure 1.

c Mde = *M. dealbata* (JX280393); Mfr = *M. fraseri* var*. pyramidata* (JX280395); Mli = *M. liliiflora* (JX280397); Mod = *M. odora* (JX280398).

d Previously reported primers (references are in Kim and Suh, 2013).

PCR was performed in a final reaction volume of 20 μL containing 1 μL of template DNA, 10 μL of 2× AmpMaster Taq (GeneAll, Seoul, Korea), 1 μL of each primer (10 μM), and 7 μL of distilled water, using a S1000 thermal cycler (BioRad, Hercules, California, USA). PCR conditions were 5 min at 95°C for pre‐denaturation, 30 cycles of 30 s at 95°C for denaturation, 30 s at 51–55°C for annealing (see Table 1), and 30 s at 72°C for extension with a final extension step of 7 min at 72°C. PCR products were checked by 1.5% agarose gel electrophoresis, stained with 0.001% ethidium bromide, and visualized under ultraviolet light using a Gel Doc XR+ System (BioRad). Each pair of primers generated 0.9–2.3 kbp of amplicons (Table 1, Fig. 1), and 27.38% of a genome overlapped with these products. The success or failure of each PCR is shown in Table 1; the overall success rate was 95%. For gap‐filling, species‐specific primers were designed outside PCR‐failed regions in each genome (data not shown). PCR products were sequenced by the Sanger method from both directions. For sequencing, PCR products were purified with a commercial purification kit (PCR SV; GeneAll) and sequenced with an ABI 3700 sequencer (Applied Biosystems, Carlsbad, California, USA). Sequence reads obtained from each PCR product were edited and aligned with Sequencher 4.9 (Gene Codes Corporation, Ann Arbor, Michigan, USA). Genome annotation was carried out with DOGMA (Wyman et al., 2004). The gene map of the chloroplast genome was created using GenomeVx (Conant and Wolfe, 2008).

Four chloroplast genomes in *Magnolia* were successfully assembled and deposited in GenBank (JX280393, JX280395, JX280397, and JX280398; Appendix S1). The size of these chloroplast genomes ranged from 158,177 to 160,070 bp. The four chloroplast genomes showed a typical circular chromosome with a quadripartite structure including inverted repeat regions ranging from 25,651 bp (*M. liliiflora*) to 26,597 bp (*M. fraseri* var. *pyramidata*), separated by large single‐copy regions ranging from 88,043 bp (*M. fraseri* var. *pyramidata*) to 88,133 bp (*M. liliiflora*) and small single‐copy regions ranging from 18,740 bp (*M. dealbata*) to 18,800 bp (*M. odora*). Overall, the gene number was 113 (79 unique genes, four rRNAs, and 30 tRNAs) in each species except for *M. liliiflora* (112 genes, 29 tRNAs). *TrnV‐*GAC is missing in the *M. liliiflora* inverted repeat region. The mean value of GC content in the four species was 39.22%.

## CONCLUSIONS

For chloroplast genome studies in *Magnolia*, we designed 250 new primers based on the chloroplast genomes of *M. kobus* and *L. tulipifera*. PCR products derived from 134 primer pairs, including 18 previously reported primers, successfully covered the entire chloroplast genomes of four *Magnolia* species from different sections within the genus. This study demonstrates that these primers will facilitate the de novo assembly of chloroplast genomes and assist with the completion of incomplete genomes.

## AUTHOR CONTRIBUTIONS

S.K. conceived and designed the project, supervised the lab and field work, and wrote the manuscript. E.S. designed the primers and completed the chloroplast genomes. S.P. wrote the first version of the manuscript.

## Supporting information


**APPENDIX S1**. Gene maps of the chloroplast genomes in (A) *Magnolia dealbata*, (B) *M. fraseri* var. *pyramidata*, (C) *M. liliiflora*, and (D) *M. odora*.

## Data Availability

Chloroplast genome sequences have been deposited at GenBank (Appendix 1), and voucher specimens for each chloroplast genome have been deposited at the herbarium of the Natural Products Research Institute (NPRI) in the Department of Pharmacology, Seoul National University (Appendix 1).

## Appendix 1 Chloroplast genome sequences used and generated in this study and their voucher information. The classification system by Figlar and Nooteboom (2004) was followed.

**Table:**

Taxa	Voucher (Herbarium)	Collection site	NCBI accession no.	Reference
Family Magnoliaceae				
Subfamily Magnolioideae				
Genus *Magnolia*				
Subgenus *Magnolia*				
Section *Rytidospermum*				
*M. dealbata*	S. Kim 1008 (NPRI)	Chollipo Arboretum, Korea	JX280393	This study
Section *Auriculata*				
*M. fraseri* var. *pyramidata*	S. Kim 1011 (NPRI)	Chollipo Arboretum, Korea	JX280395	This study
Subgenus *Yulania*				
Section *Yulania*				
*M. kobus*	—		NC_023237	Song et al., 2018
*M. liliiflora*	S. Kim 1014 (NPRI)	Chollipo Arboretum, Korea	JX280397	This study
Section *Michelia*				
*M. odora*	S. Kim 1099 (NPRI)	South China Botanical Garden, China	JX280398	This study
Subfamily Liriodendroidae				
Genus *Liriodendron*				
*L. tulipifera*	—		NC_008326	Cai et al., 2006
