# Supplementary figures and images for: Primers for complete chloroplast genome sequencing in Magnolia
Source: Appl Plant Sci. 2019 Sep 19;7(9):e11286. doi: 10.1002/aps3.11286 (PMC6764489; doi:10.1002/aps3.11286)

# B

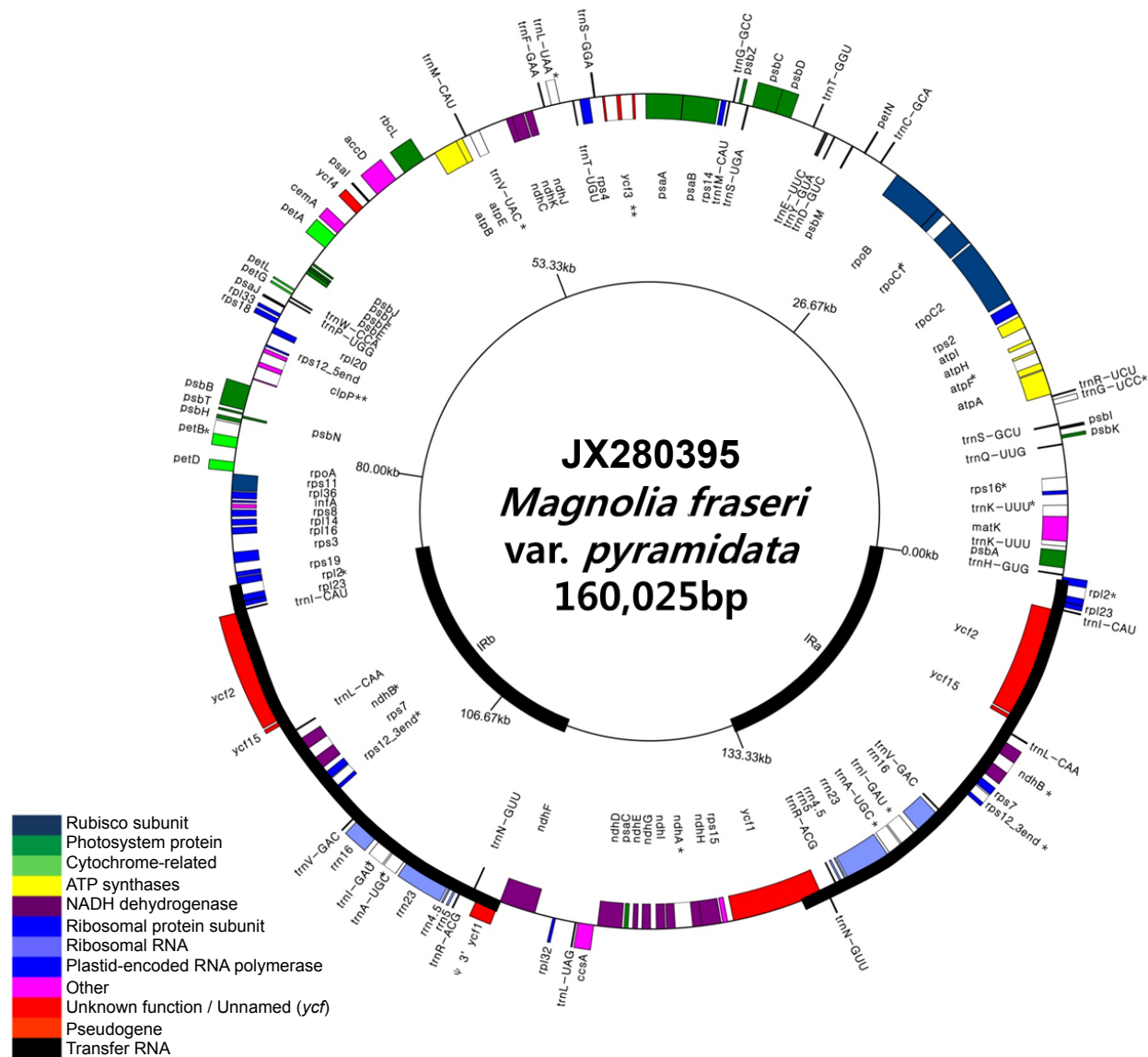

C

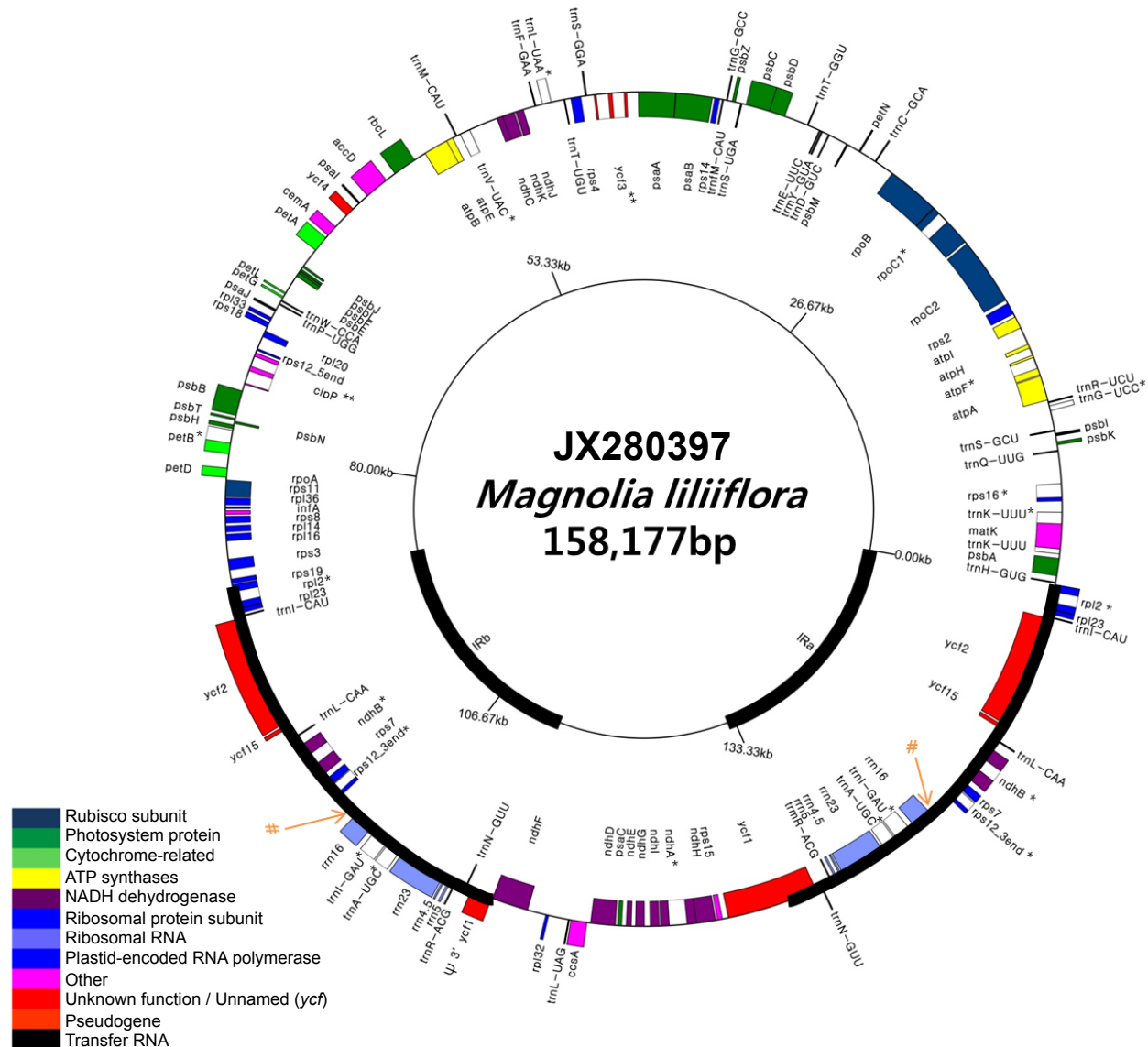

D

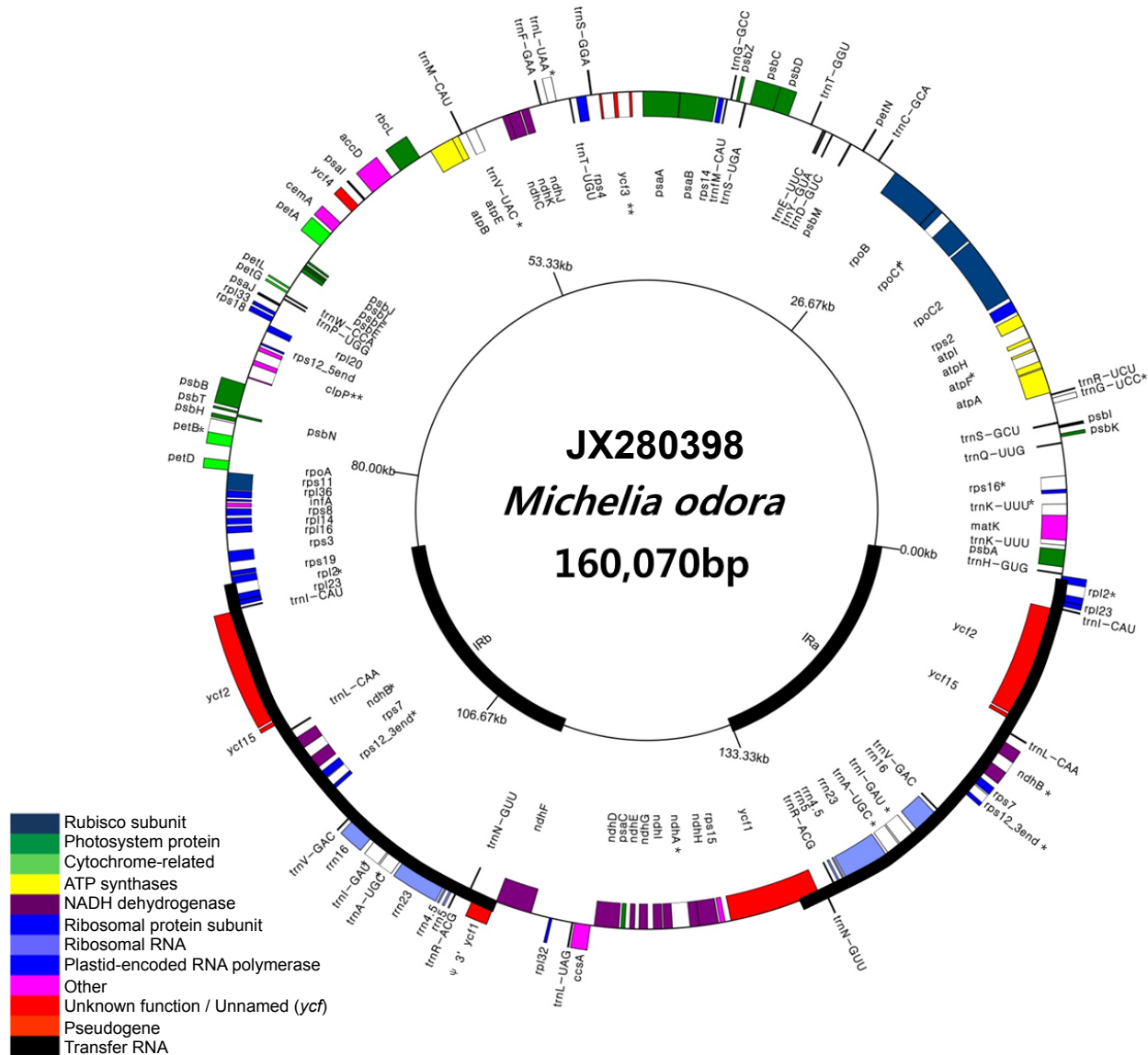

Supplement: Supplementary file 1 — APPENDIX S1. Gene maps of the chloroplast genomes in (A) Magnolia dealbata, (B) M. fraseri var. pyramidata, (C) M. liliiflora, and (D) M. odora. [file APS3-7-e11286-s001.pdf]
